# Relationships between the Content of Phenolic Compounds and the Antioxidant Activity of Polish Honey Varieties as a Tool for Botanical Discrimination

**DOI:** 10.3390/molecules26061810

**Published:** 2021-03-23

**Authors:** Monika Kędzierska-Matysek, Małgorzata Stryjecka, Anna Teter, Piotr Skałecki, Piotr Domaradzki, Mariusz Florek

**Affiliations:** 1 Institute of Quality Assessment and Processing of Animal Products, University of Life Sciences in Lublin, Akademicka 13, 20-950 Lublin, Poland; monika.matysek@up.lublin.pl (M.K.-M.); anna.wolanciuk@up.lublin.pl (A.T.); piotr.skalecki@up.lublin.pl (P.S.); piotr.domaradzki@up.lublin.pl (P.D.); 2 Institute of Agricultural Sciences, State School of Higher Education in Chełm, Pocztowa 54, 22-100 Chełm, Poland; mstryjecka@pwsz.chelm.pl

**Keywords:** honey, botanical origin, phenolic compounds, antioxidant activity

## Abstract

The study compared the content of eight phenolic acids and four flavonoids and the antioxidant activity of six Polish varietal honeys. An attempt was also made to determine the correlations between the antioxidant parameters of the honeys and their polyphenol profile using principal component analysis. Total phenolic content (TPC), total flavonoid content (TFC), antioxidant activity (ABTS) and reduction capacity (FRAP) were determined spectrophotometrically, and the phenolic compounds were determined using high-performance liquid chromatography (HPLC). The buckwheat honeys showed the strongest antioxidant activity, most likely because they had the highest concentrations of total phenols, total flavonoids, p-hydroxybenzoic acid, caffeic acid, p-coumaric acid, vanillic acid and chrysin. The principal component analysis (PCA) of the data showed significant relationships between the botanic origin of the honey, the total content of phenolic compounds and flavonoids and the antioxidant activity of the six Polish varietal honeys. The strongest, significant correlations were shown for parameters of antioxidant activity and TPC, TFC, p-hydroxybenzoic acid, caffeic acid and p-coumaric acid. Analysis of four principal components (explaining 86.9% of the total variance), as a classification tool, confirmed the distinctiveness of the Polish honeys in terms of their antioxidant activity and content of phenolic compounds.

## 1. Introduction

Honey is a natural food product made by bees from plant nectar and/or honeydew with an admixture of substances specific to these insects. In addition to nutrients, honey contains bioactive substances that can exert a positive effect on the human body [1]. The health-promoting properties of honey are due to the presence of phenolic compounds, including flavonoids, phenolic acids and their esters, as well as organic acids, free amino acids, vitamins (E—tocopherol; C—ascorbic acid), carotenoid derivatives, enzymes and bioelements (Zn, Cu, Se, Mn and Co). These compounds exhibit antioxidant properties, actively protecting biological compounds against oxidation [2].

Oxidation processes can be counteracted through the use of antioxidant agents. Antioxidants can prevent numerous diseases or even alleviate their symptoms [2]. Unfortunately, their content in the body is often insufficient. Bee products can therefore be supplementary sources of antioxidants in a balanced diet [3]. The antioxidant capacity of honey depends primarily on its botanic origin, which in turn depends on the climate zone [4]. The complex composition of honey, the interactions between various antioxidant compounds and potential synergistic relationships between them can also play an important role in total antioxidant capacity [5].

One important group of biologically active molecules in honey is polyphenols [6], which have a broad spectrum of biological activity and are natural antioxidants [7]. Phenolic compounds, including phenolic acids, are secondary metabolites of plants and fungi that protect against environmental factors such as UV radiation, viruses and bacteria.

Due to the health-promoting activity of honey, determined mainly by its antioxidant properties, it should be a significant component of the human diet [8]. The presence of phenolic compounds in honey and their role and importance in antioxidant protection in humans have prompted detailed research on this topic. Many scientific studies focus on detection of phenolic compounds in honey—determination of their concentration or absence. This can be a means of constructing a profile of these compounds in varietal honeys from various geographic areas and, in consequence, can serve as a tool for classifying monofloral varieties [9].

Multivariate analysis (e.g., principal component analysis-PCA; hierarchical cluster analysis-HCA; linear discriminate analysis-LDA, and others) has very often been used to evaluate and/or classify honeys in terms of their chemical composition or their physicochemical or biological properties. Numerous papers have confirmed the suitability of this method for honey, used either alone [10,11,12,13,14] or in combination with spectroscopic techniques [15].

The study compared the content of phenolic acids and flavonoids and the antioxidant activity of Polish varietal honeys. An attempt was also made to determine the correlations between the antioxidant parameters of the honeys and their polyphenol profile using principal component analysis (PCA)/multivariate analysis.

## 2. Results and Discussion

### 2.1. Phenolic Acids

Seven phenolic acids were identified in all of the honeys tested: p-hydroxybenzoic, vanillic, syringic, p-coumaric, ferulic, benzoic and cinnamic (Table 1). Caffeic acid (as the eighth acid) was found in the buckwheat (0.54 mg/kg), multifloral (0.23 mg/kg) and linden (0.15 mg/kg) honeys. The buckwheat honey significantly had (*p* ≤ 0.01) the highest content of p-hydroxybenzoic (13.69 mg/kg), p-coumaric (7.04 mg/kg), vanillic (1.86 mg/kg), caffeic (0.54 mg/kg) and cinnamic (0.38 mg/kg) acids among all varieties. In the remaining varieties, the phenolic acids present in the highest amounts were benzoic acid (from 3.58 in rapeseed honey to 15.67 mg/kg in multifloral honey) and p-coumaric acid (from 1.39 in multifloral honey to 3.30 mg/kg in acacia honey). Acacia honey contained the most ferulic acid (2.59 mg/kg), and rapeseed honey contained the most syringic acid (0.71 mg/kg).

The phenol composition of honey depends primarily on its botanic origin [2], and the quantity of phenolic compounds can vary depending on the season of the year, climatic conditions and processing factors [16]. Comparison of results obtained for the polyphenol composition of honey can be difficult due to its complex matrix, the low concentrations of these compounds and differences in their analysis and presentation [7]. The results presented here are consistent with those obtained by Wilczyńska [17], who noted the highest content of p-hydroxybenzoic acid (12.99 mg/kg) and p-coumaric acid (6.20 mg/kg) in buckwheat honey. In addition, irrespective of the variety, the phenolic acids detected most often and in the highest quantities, apart from p-hydroxybenzoic acid, were syringic, p-coumaric and ferulic acids, whose content was significantly dependent on the botanic origin of the honey (except for syringic acid). Among the honey varieties, buckwheat and heather stood out in terms of content of phenolic acids. Similarly, Socha et al. [18] reported the highest content of phenolic acids, including caffeic acid, in buckwheat honey. Not all varieties tested were shown to contain sinapic, chlorogenic and ferulic acids, while the dominant phenolic acids were p-coumaric and gallic acids. The differences in comparison to the present study may have been due to analysis of different phenolic acids.

In Romanian honey, 12 phenolic compounds were detected, i.e., vanillic acid, caffeic acid, p-coumaric acid, quercetin and kaempferol [19], as well as others (4-hydroxybenzoic acid, gallic acid, protocatechuic acid, chlorogenic acid, rosmarinic acid, myricetin and luteolin) that were not identified in the present study. The content of these compounds, however, varied depending on the variety. In honey from Romania, the highest content of myricetin was noted in rape, thyme and polyfloral honeys (22.3 mg/kg, 19.9 mg/kg, and 17.3 mg/kg, respectively), the highest content of vanillic acid in mint and sunflower honeys (30.3 mg/kg and 23.5 mg/kg) and the highest content of protocatechuic acid (25.7 mg/kg) and 4-hydroxybenzoic acid (23.3 mg/kg) in raspberry honey [16]. Rosmarinic acid was detected only in raspberry honey (3 mg/kg) and kaempferol (3.8 mg/kg) only in polyfloral honey, while luteolin was not detected at all.

Italian multifloral honeys contained much more caffeic acid (13.83 mg/kg) than honey of this variety in the present study (0.23 mg/kg), which in turn contained more benzoic acid (15.68 mg/kg) than the multifloral honeys from Italy (0.56 mg/kg) [20]. In ethyl acetate extracts of buckwheat honey from Asia, the dominant phenolic acids are p-hydroxybenzoic acid (50.3 μg/mg), p-coumaric acid (11.0 μg/mg) and chlorogenic acid (29.5 μg/mg) [21], which were not analyzed in the present study. A similar level of p-coumaric acid (12.52 mg/kg) was reported in buckwheat honey [22].

### 2.2. Flavonoids

Four flavonoids were identified in the varietal honeys from Poland: quercetin, apigenin, kaempferol and chrysin (Table 2). Quercetin was present in the highest concentrations in multifloral (11.33 mg/kg), buckwheat (2.51 mg/kg), linden (1.72 mg/kg) and acacia (0.86 mg/kg) honeys, but was not found in honeydew honey. Chrysin was dominant in buckwheat honey (0.99 mg/kg), kaempferol in rapeseed honey (0.81 mg/kg) and apigenin in honeydew honey (0.41 mg/kg). Apigenin was not found in linden honey.

The results are consistent with those reported by Wilczyńska [17] for quercetin in Polish varietal honeys. Similarly, Socha et al. [18] detected the highest content of kaempferol in rapeseed honey and the highest content of chrysin in buckwheat honey. The flavonoid present in the highest amounts in buckwheat honey from Asia was rutin (35.94 mg/kg), while the content of hesperetin was somewhat lower (23.76 mg/kg) [22]. Quercetin (1.52 μg/mg) and kaempferol (1.47 μg/mg) were the main flavonoids in extracts of buckwheat honey from Asia, as they were present in it in the highest amounts [21]. The content of individual flavonoids depends not only on the variety but also on geographic origin [23]. Moreover, organic honey contained significantly more chrysin than conventional honey. Further, other flavonoids are present in acacia honeys (genistein, galangin, luteolin, myricetin, pinobanksin and pinocembrin) [2].

### 2.3. Antioxidant Activity and Phenolic Compounds

Among the varieties tested, buckwheat honey had the highest total concentration of phenolic compounds (567.9 mg GAE/kg) and flavonoids (27.6 mg QE/kg) and, in consequence, the highest antioxidant potential, expressed as ABTS (6.5 mM TE/100 g) and FRAP (2144.3 µm Fe(II)/kg) (Table 3). The content of phenolic compounds and flavonoids in buckwheat honey was much higher (at least two–three times) than in the other varieties. In terms of antioxidant activity, the Polish honey varieties can be ordered as follows: buckwheat > honeydew > multifloral > linden > rapeseed and acacia.

The content of phenolic compounds in Polish buckwheat honey [12] was twice as high as in the present study, while the TPC in rapeseed, linden and acacia honeys was comparable to the values obtained in our study. Low total polyphenol content in rapeseed and acacia honeys was previously noted in Romanian honeys [19]. In buckwheat honey from China, the total content of phenols was three times as high as in manuka honey, amounting to 1498 mg/kg. A high total phenolic content was reported in buckwheat honey (2040 mg GA/kg) [22]. A comparable range of total polyphenol content (152.9–321.8 mg GAE/kg) to that obtained in the present study (except for buckwheat honey) was reported for retail honey in Mexico [24].

The total polyphenol content in the present study ranged from 158.4 to 567.9 mg/kg. A lower range has been reported for honey from Slovenia (44.8–241.4 mg/kg) [4], Romania (120.0–260.5 mg/kg) [25], Italy (108.2–146.7 mg/kg) [26] and Portugal (198.5–214.3 mg/kg) [27]. Higher content was found in honey from Spain (339–1542 mg/kg) [28], Brazil (611.1–1753.9 mg/kg) [29], Italy (165–1333 mg/kg) [30], Sudan (559.7 and 2249.9 mg/kg) [31] and Turkey (343.7 and 4707 mg/kg) [32]. In Spanish honeys, as many as 49 phenolic compounds were detected, of which 46 were quantified [5]. They observed significant (*p* < 0.001) variation between samples in the content of total phenolic compounds (from 231 to 1580 mg/kg) and total flavonoid content (from 165 to 593 mg/kg).

In the present study, the highest total flavonoid content was found in the buckwheat (27.6 mg QE/kg) and honeydew (20.6 mg QE/kg) honeys and the lowest in the rapeseed (2.2 mg QE/kg) and acacia (1.6 mg QE/kg) honeys (Table 3). For various honeys from Poland, the average flavonoid content expressed as quercetin equivalent ranged from 0.14 to 29.94 mg/kg [17]. The present study confirms previous observations by Wieczorek et al. [33], who found the highest flavonoid concentration in buckwheat honey (23.4 mg/kg and 32.3 mg/kg), followed by linden (4.3–15.1 mg/kg) and multifloral (1.8–14.4 mg/kg), and the lowest in acacia honey (1.1 mg/kg).

The world literature also reports varied total flavonoid content in honey from different countries. In honey from Sudan, the concentrations of these compounds ranged from 14.3 to 298.1 mg GAE/kg [31], and in Romanian honey from 10.8 to 32 mg QE/kg [25]. These ranges are comparable to those obtained in the present study. In Spanish honey, the total flavonoid content ranged from 14 to 103 mg QE/kg [28], in Brazilian honey from 21.6 to 109.1 mg QE/kg [29], in Italian honey from 50.9 to 140.5 mg QE/kg [26] and in Portuguese honey from 117 to 135 mg/kg [27].

Among the honey varieties, the highest antioxidant activity was found in the buckwheat honey and the lowest in the rapeseed and acacia honeys (Table 3). Similar observations were previously reported by Socha et al. [18], who obtained the highest antioxidant activity, measured in reactions with DPPH and ABTS, for buckwheat honey and the lowest for rapeseed honey. According to Piszcz and Głód [34], based on an evaluation of total antioxidant potential (TAP), varietal honeys can be ordered as follows: buckwheat > honeydew > linden > multifloral > acacia. Cheng et al. [22] ascribe hepatoprotective activity and inhibition of DNA damage to buckwheat honey, primarily due to its high antioxidant capacity. For Czech and Slovakian honeys in terms of their total antioxidant capacity (TAC), measured using the ABTS reagent, and their content of polyphenols (PP), the following order was determined: honeydew > multi flower > forest > floral honeys > rape > acacia [35]. The TAC (ABTS) values in these honeys ranged from 155 to 896 mg TE/kg, and polyphenol content ranged from 540 to 2542 mg GAE/kg. Italian honeys exhibited radical scavenging capacity from 192 to 2703 μmol TE/kg in the ABTS test [30]. The antioxidant activity of Brazilian honey measured by the ABTS method ranged from 701 to 7006 μmol TE/kg, and from 662.8 to 3885 μmol Fe(II)/kg in the FRAP test [29].

### 2.4. Correlations

Ferric reducing antioxidant power (FRAP) and radical scavenging activity (ABTS) were generally positively and significantly correlated with the concentrations of individual polyphenols (except for benzoic and ferulic acids) and their total content (Table 4). Parameters of antioxidant activity (ABTS and FRAP) were also positively correlated with the level of apigenin and chrysin and with total flavonoid content (Table 5). ABTS and FRAP were most strongly (*p* ≤ 0.001) and positively correlated with total phenol content (TPC) (0.724 ≤ r ≤ 0.885), p-hydroxybenzoic acid (0.672 ≤ r ≤ 0.819), caffeic acid (0.757 ≤ r ≤ 0.781) and p-coumaric acid (0.466 ≤ r ≤ 0.690), and least strongly with vanillic acid (0.313 ≤ r ≤ 0.466) and syringic acid (0.310 ≤ r ≤ 0.330). In the case of flavonoids, the strongest correlations were obtained for total flavonoid content (TFC) with ABTS and FRAP (0.614 ≤ r ≤ 0.730). In addition, apigenin was correlated with FRAP (r = 0.514) and chrysin with ABTS (r = 0.455).

The correlation coefficients obtained in the present study are similar to those reported for antioxidant activity and TPC (total phenol content) or TFC (total flavonoid content) (0.919, *p* < 0.0001), or the content of polyphenolic compounds (0.843 ≤ r ≤ 0.956, *p* < 0.0001) [30]. Many authors have confirmed strong relationships between high content of polyphenols in honey and total antioxidant capacity (ABTS; r = 0.9005) [35], total phenol content and total antioxidant activity (rPC/FRAP = 0.885) [36] or TP and FRAP (r = 0.9751) [12]. Lower correlation coefficients were reported by Perna et al. [26]. ABTS was significantly (*p* ≤ 0.001) correlated with TFC (r = 0.61) and with TPC (r = 0.48). The correlation coefficient was r = 0.66 for FRAP with TFC and r = 0.36 for FRAP with TPC. The higher correlation between FRAP and total flavonoid content than between FRAP and total phenol content suggests that the reducing power of honey is associated with flavonoids, which reduce Fe^+3^ to Fe^+2^ [26].

The stronger antioxidant activity of dark honeys is due to their higher content of phenolic compounds than in light honeys [8]. This is supported by the results of the present study for the dark buckwheat honey. However, some authors point out that while phenolic compounds can play an important role in antioxidant activity, other non-phenolic antioxidants (e.g., proteins, ascorbic acid and catalase) may contribute to the whole pattern of antioxidant activity [26].

### 2.5. Principal Component Analysis

The results obtained for antioxidant activity and content of polyphenols and flavonoids in the Polish honey varieties were further analyzed by principal component analysis (PCA). The data were subjected to a multivariate approach using 7 variables and 64 samples. Four principal components with eigenvalues exceeding 1 (Kaiser criterion) explained 86.90% of the total variance, with PC1 accounting for 43.12%, PC2 for 20.98%, PC3 for 15.38% and PC4 for 7.41% (Table 5).

**Figure 1:**
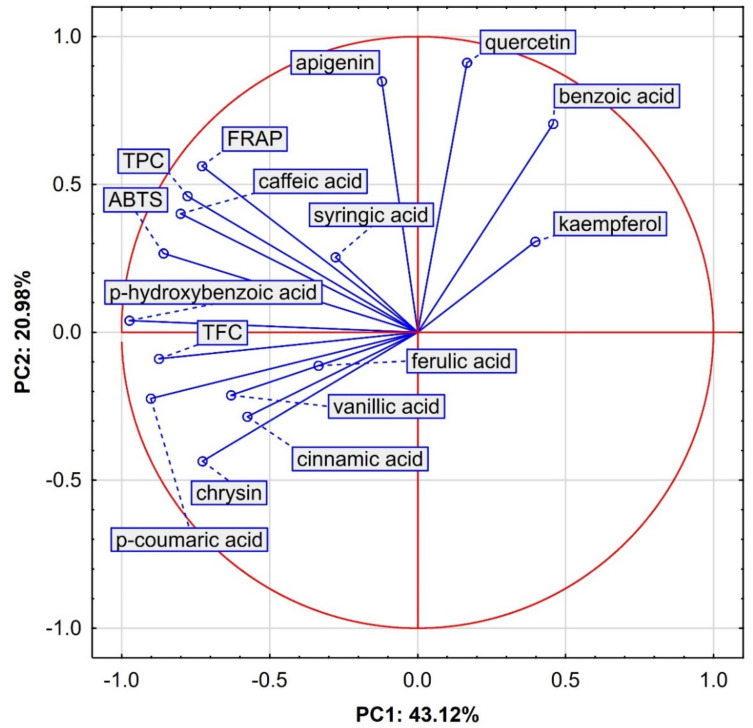
Projection of variables in a two-factor plane (PC1 × PC2); ABTS-antioxidant capacity; FRAP-reduction capacity; TPC-total polyphenols content; TFC-total flavonoids content.

Figure 1 visualizes the projection of variables as a two-factor plane (PC1 × PC2). The first component (PC1), explaining 43.12% of the total variance, has a negative correlation with most variables, including p-hydroxybenzoic acid (−0.974), p-coumaric acid (−0.902), total flavonoid content (−0.874), ABTS (−0.859), caffeic acid (−0.801), total polyphenol content (−0.778), FRAP (−0.728), chrysin (−0.727) and cinnamic acid (−0.576) (Table 6). The second component (PC 2), explaining 20.98% of the total variance, has a positive correlation with quercetin (0.912), apigenin (0.849) and benzoic acid (0.705). The third component (PC3), explaining 15.38% of the total variance, has a negative correlation with syringic acid (−0.748), vanillic acid (−0.674) and kaempferol (−0.600), while the fourth component (responsible for 7.41% of the total variance) has a positive correlation only with ferulic acid (0.848).

**Figure 2:**
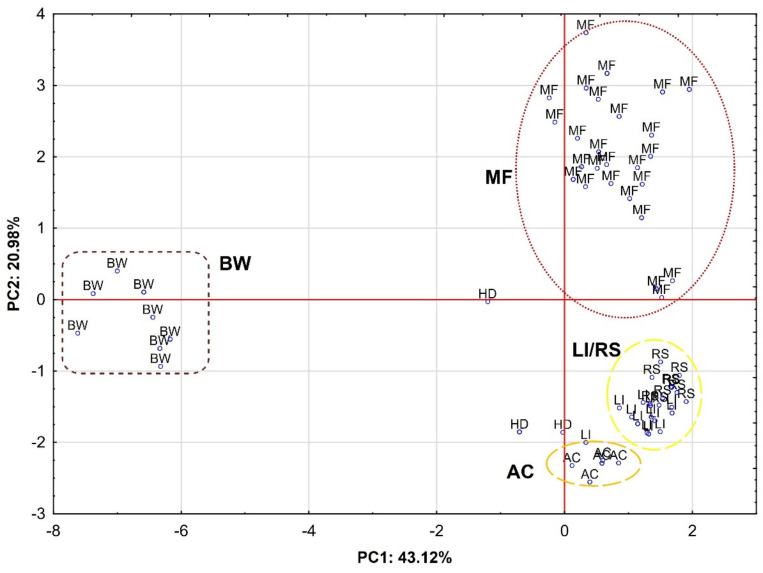
Projection of cases depending on the botanical origin of the honey in a two-factor plane (PC1 × PC2); ABTS-antioxidant capacity; FRAP-reduction capacity; honey variety: RS-rapeseed, BW-buckwheat, LI-linden, AC-black locust, MF-multifloral, HD-honeydew.

Figure 2 shows the projection of cases of honey samples in the coordinate system defined by PC1 × PC2. There is a clear separation of four groups depending on the botanic origin.

The first group, located on the left side of the plot, consists of buckwheat honey samples (BW), which means that they have negative values for PC1 and, in most cases (five samples), negative values for PC2. Therefore, the buckwheat honey located in this area of the plot showed the highest values for antioxidant activity (FRAP and ABTS), TPC, TFC, p-hydroxybenzoic acid and chrysin. Among the other honey varieties, the second group is composed of multifloral samples (MF) in the upper right square of the plot, which is positively correlated first with PC2 and then with PC1. This area represents the highest values for quercetin, apigenin and benzoic acid, which clearly corresponds to the results in Table 1 for MF honey. The third group is a combination of samples of rapeseed (RS) and linden (LI) honeys and, together with the fourth group, composed of acacia honey, is located in the lower right square of the plot. In contrast, the honeydew samples (HD) were more scattered, but they were generally negatively correlated with both components (PC1 and PC2). Summing up, the data presented in Figure 1 and Figure 2 confirm the results given in Table 1 and Table 2. Buckwheat honey showed the highest antioxidant activity in connection with the highest total content of flavonoids and polyphenol compounds.

## 3. Materials and Methods

### 3.1. Sampling

Six popular Polish honey varieties were selected for the study: multifloral (MF, *n* = 27), linden (LI, *n* = 13), rapeseed (RS, *n* = 10), buckwheat (BW, *n* = 8), black locust/acacia (AC, *n* = 5) and honeydew (HD, *n* = 3). The honeys were purchased directly from beekeepers, whose apiaries were located in south-eastern Poland (Lublin region). All of the honeys were produced in 2019 and were sold in glass jars, each sold as a separate item. Their origin was confirmed by pollen analysis [37].

### 3.2. Chemical Analyses

Sample extraction was carried out using solutions of ethanol (96%, Avantor-POCh, Gliwice, Poland) and water by dissolving 3 g of the honey sample (with an accuracy of 0.001 g) in a mixture of ethanol and water (50:50 *v*/*v*).

Total flavonoid content (TFC) was determined in a reaction with aluminum chloride [38]. A calibration curve was plotted for a standard quercetin solution (QE, Sigma-Aldrich, Munich, Germany) in a range of concentrations from 100 to 500 µg/mL. Absorbance was measured on a UV-2600i spectrophotometer (Shimadzu, Tokyo, Japan) at λ = 510 nm. Total flavonoid content was expressed as quercetin equivalent (QE) in mg QE/100 g of sample.

Total phenolic content (TPC) was determined in a reaction with Folin–Ciocalteu (F–C) reagent [39]. A calibration curve was plotted for standard solution of gallic acid (Sigma-Aldrich, Munich, Germany) in a range of concentrations from 0 to 100 µg/mL. Absorbance was measured on a UV-2600i spectrophotometer (Shimadzu, Tokyo, Japan) at λ = 760 nm. The total content of polyphenols was expressed as gallic acid equivalent in mg GAE/kg of honey.

The polyphenol profile was determined using high-performance liquid chromatography (HPLC) with an AZURA UHPLC liquid chromatograph system (Knauer, Berlin, Germany). Phenolic compounds were extracted using ethyl acetate [18]. The analysis was carried out on a Purospher RP-18 column (250 × 4 mm, 5 µm, Merck, Darmstadt, Germany) at 30 °C and a flow speed of 1 mL/min. Qualitative analysis of phenolic compounds was performed by comparing the UV spectra obtained for the test compounds with the spectra for phenol standards using a DAD detector. Quantitative analysis of phenolic compounds was based on calibration curves plotted separately for each standard (flavonoids and phenolic acids, Sigma-Aldrich, Munich, Germany).

Antioxidant activity was determined in a reaction with the ABTS radical cation (Sigma-Aldrich, Munich, Germany) [40]. A calibration curve was plotted in a range of concentrations from 0 to 0.09 µM using Trolox as a standard (Sigma-Aldrich, Munich, Germany). Absorbance was measured on a UV-2600i spectrophotometer (Shimadzu, Tokyo, Japan) at λ = 734 nm. The results were expressed in mM of Trolox per 100 g of sample (mM TE/100 g).

Reduction capacity was determined by the FRAP method [41] with modification [4]. The FRAP reagent contained 2.5 mL 10 mM of TPTZ solution (Sigma Aldrich, Munich, Germany) in 40 mM HCl, 2.5 mL 20 mM FeCl_3_ (POCH) and 25 mL 0.3 M acetic buffer (pH = 3.6). The test sample contained 0.2 mL of honey and 1.8 mL of FRAP reagent. Absorbance was measured on a UV-2600i spectrophotometer (Shimadzu, Tokyo, Japan) at λ = 593 nm, following 10 min incubation at 37 °C. The results were expressed in µmol TE/kg.

### 3.3. Statistical Analysis

Statistical analysis of the results was performed in Statistica ver. 13 (TIBCO Software Inc., Palo Alto, CA, USA). One-way analysis of variance (ANOVA) followed by Tukey’s (HSD) test was used to compare mean contents of phenolic acids and flavonoids with parameters of the antioxidant activity of the honey varieties (multifloral, rapeseed, buckwheat, linden, acacia and honeydew). Differences between means at confidence levels of 95% and 99% (*p* ≤ 0.05 and *p* ≤ 0.01, respectively) were considered statistically significant. The mean and standard deviation are presented in the tables. The relationships between parameters of antioxidant activity (FRAP and ABTS) and phenolic acids and flavonoids in honeys were determined by calculating Pearson’s correlation coefficients. In order to demonstrate the diversity among honey varieties, the data were further verified by principal component analysis (PCA).

## 4. Conclusions

The research and principal component analysis (PCA) of the data showed significant relationships between the botanic origin of the honey, the total content of phenolic compounds and flavonoids and the antioxidant activity of the six Polish varietal honeys. The buckwheat honeys showed the strongest antioxidant activity, most likely because they had the highest concentrations of total phenols, total flavonoids, p-hydroxybenzoic acid, caffeic acid, p-coumaric acid, vanillic acid and chrysin. The strongest, significant correlations were shown for parameters of antioxidant activity and TPC, TFC, p-hydroxybenzoic acid, caffeic acid and p-coumaric acid. Analysis of four principal components (explaining 86.9% of the total variance), as a classification tool, confirmed the distinctiveness of the Polish honeys in terms of their antioxidant activity and content of phenolic compounds.

## Figures and Tables

**Table 1:** Content of phenolic acids (mg/kg) in Polish varietal honeys.

Phenolic Acid	Honey Variety						*p*
	Multifloral (MF)	Rapeseed (RS)	Buckwheat (BW)	Linden (LI)	Acacia (AC)	Honeydew (HD)	
p-hydroxybenzoic	2.08 ± 0.43 ^B^	0.98 ± 0.15 ^A^	13.69 ± 0.83 ^C^	0.78 ± 0.08 ^A^	2.37 ± 0.16 ^B^	1.89 ± 0.65 ^B^	0.000
caffeic	0.23 ± 0.04 ^C^	0.00 ^A^	0.54 ± 0.06 ^D^	0.15 ± 0.02 ^B^	0.00 ^A^	0.00 ^A^	0.000
vanillic	0.95 ± 0.16 ^B^	1.49 ± 0.25 ^C^	1.86 ± 0.16 ^D^	0.69 ± 0.06 ^A^	1.50 ± 0.10 ^C^	1.61 ± 0.34 ^C, D^	0.000
syringic	0.46 ± 0.12 ^B^	0.71 ± 0.18 ^D^	0.61 ± 0.04 ^C^	0.22 ± 0.03 ^A^	0.24 ± 0.04 ^A^	0.55 ± 0.08 ^B, C^	0.000
p-coumaric	1.39 ± 0.21 ^A^	2.67 ± 0.44 ^B^	7.04 ± 0.37 ^D^	1.18 ± 0.10 ^A^	3.30 ± 0.18 ^C^	2.44 ± 0.39 ^B^	0.000
ferulic	0.92 ± 0.15 ^C^	0.70 ± 0.08 ^B^	1.34 ± 0.21 ^D^	0.42 ± 0.05 ^A^	2.59 ± 0.17 ^F^	1.59 ± 0.31 ^E^	0.000
benzoic	15.67 ± 2.23 ^C^	3.58 ± 0.37 ^A, B^	2.59 ± 0.25 ^A^	11.53 ± 0.39 ^C^	5.40 ± 0.22 ^B^	4.45 ± 0.56 ^A, B^	0.000
cinnamic	0.17 ± 0.03 ^B^	0.08 ± 0.01 ^A^	0.38 ± 0.11 ^D^	0.29 ± 0.06 ^C^	0.32 ± 0.03 ^C, D^	0.15 ± 0.04 ^A, B^	0.000

Means with different letters (**^A^**^, **B**, **C**, **D**, **E**^) in rows differ significantly (*p* ≤ 0.01).

**Table 2:** Content of flavonoids (mg/kg) in Polish varietal honeys.

Flavonoid	Honey Variety						*p*
	Multifloral (MF)	Rapeseed (RS)	Buckwheat (BW)	Linden (LI)	Acacia (AC)	Honeydew (HD)	
quercetin	11.33 ± 3.78 ^B^	0.74 ± 0.09 ^A^	2.51 ± 0.26 ^A^	1.72 ± 0.27 ^A^	0.86 ± 0.07 ^A^	0.00 ^A^	0.000
apigenin	0.58 ± 0.14 ^C^	0.28 ± 0.03 ^B^	0.38 ± 0.05 ^B^	0.00 ^A^	0.26 ± 0.03 ^B^	0.41 ± 0.14 ^B, C^	0.000
kaempferol	0.59 ± 0.17 ^C^	0.81 ± 0.06 ^D^	0.39 ± 0.02 ^B^	0.42 ± 0.06 ^B^	0.57 ± 0.02 ^C^	0.20 ± 0.06 ^A^	0.000
chrysin	0.27 ± 0.06 ^A^	0.38 ± 0.05 ^B^	0.99 ± 0.05 ^D^	0.62 ± 0.16 ^C^	0.45 ± 0.02 ^B^	0.25 ± 0.08 ^A^	0.000

Means with different letters (**^A^**^, **B**, **C**, **D**^) in rows differ significantly (*p* ≤ 0.01).

**Table 3:** Antioxidant activity of honey from Poland.

Parameter	Honey Variety						*p*
	Multifloral (MF)	Rapeseed (RS)	Buckwheat (BW)	Linden (LI)	Acacia (AC)	Honeydew (HD)	
Total flavonoids (mg QE/kg)	5.7 ± 2.06 ^B^	2.2 ± 0.11 ^A^	27.6 ± 3.93 ^E^	9.0 ± 3.31 ^C^	1.6 ± 0.30 ^A^	20.6 ± 0.91 ^D^	0.000
Total polyphenols (mg GAE/kg)	328.9 ± 90.29 ^C^	158.4 ± 23.03 ^A^	567.9 ± 101.92 ^D^	224.3 ± 41.69 ^B^	187.0 ± 34.30 ^A, B^	164.3 ± 2.54 ^A, B^	0.000
ABTS (mM TE/100 g)	2.9 ± 1.17 ^B^	1.7 ± 0.08 ^A^	6.5 ± 0.95 ^C^	1.9 ± 0.34 ^A^	1.8 ± 0.63 ^A^	3.8 ± 0.63 ^B^	0.000
FRAP (µM Fe(II)/kg)	1279.9 ± 493.78 ^B^	466.7 ± 106.45 ^A^	2144.3 ± 280.69 ^C^	533.1 ± 129.46 ^A^	288.6 ± 25.52 ^A^	1359.4 ± 472.96 ^B^	0.000

Means with different letters (**^A^**^, **B**, **C**, **D**, **E**^) in rows differ significantly (*p* ≤ 0.01).

**Table 4:** Pearson’s correlation.

Parameter	ABTS	FRAP
p-hydroxybenzoic acid	0.819 ***	0.672 ***
caffeic acid	0.757 ***	0.781 ***
vanillic acid	0.466 ***	0.313 **
syringic acid	0.330 **	0.310 *
p-coumaric acid	0.690 ***	0.466 ***
ferulic acid	0.229	0.142
benzoic acid	−0.229	0.062
cinnamic acid	0.366 **	0.210
quercetin	0.028	0.288 *
apigenin	0.307 *	0.514 ***
kaempferol	−0.331 **	−0.230
chrysin	0.455 ***	0.242
Total polyphenols content	0.724 ***	0.885 ***
Total flavonoids content	0.730 ***	0.614 ***

N = 66, * *p* ≤ 0.05, ** *p* ≤ 0.01, *** *p* ≤ 0.001.

**Table 5:** Eigenvalues and the proportion of variation (%) explained by the principal components.

Component	Eigenvalue	Proportion	Cumulative
1	6.90	43.12	43.12
2	3.36	20.98	64.10
3	2.46	15.38	79.49
4	1.19	7.41	86.90
5	0.73	4.58	91.49
6	0.34	2.15	93.64
7	0.22	1.35	94.99
8	0.20	1.23	96.23
9	0.17	1.07	97.29
10	0.13	0.81	98.11
11	0.11	0.67	98.76
12	0.08	0.49	99.26
13	0.06	0.36	99.62
14	0.03	0.19	99.81
15	0.02	0.14	99.95
16	0.01	0.05	100.00

**Table 6:** Correlations between the principal components and the original variables.

Variable	Principal Component			
	1	2	3	4
p-hydroxybenzoic acid	−0.974	0.040	−0.043	−0.016
caffeic acid	−0.801	0.401	0.333	−0.135
vanillic acid	−0.630	−0.213	−0.674	0.118
syringic acid	−0.278	0.253	−0.748	−0.387
p-coumaric acid	−0.902	−0.224	−0.297	0.020
ferulic acid	−0.336	−0.113	−0.347	0.848
benzoic acid	0.458	0.705	0.484	0.082
cinnamic acid	−0.576	−0.285	0.508	0.338
Total polyphenols content	−0.778	0.461	0.192	−0.062
quercetin	0.167	0.912	0.105	0.114
apigenin	−0.121	0.849	−0.354	0.240
kaempferol	0.397	0.306	−0.600	−0.147
chrysin	−0.727	−0.436	0.237	−0.228
Total flavonoids content	−0.874	−0.089	0.202	−0.120
FRAP	−0.728	0.562	0.057	−0.034
ABTS	−0.859	0.267	0.004	−0.031

## Data Availability

Data sharing not applicable.

## Sample Availability

Samples of the compounds are not available from the authors.
